# Assessment of the effect of addition of 24 hours of oral tranexamic acid post-operatively to a single intraoperative intravenous dose of tranexamic acid on calculated blood loss following primary hip and knee arthroplasty (TRAC-24): a study protocol for a randomised controlled trial

**DOI:** 10.1186/s13063-018-2784-3

**Published:** 2018-07-31

**Authors:** Janet Hill, Paul Magill, Alastair Dorman, Rosemary Hogg, Andrew Eggleton, Gary Benson, Margaret McFarland, Lynn Murphy, Evie Gardner, Leeann Bryce, Una Martin, Catherine Adams, Jennifer Bell, Christina Campbell, Ashley Agus, Glenn Phair, Dennis Molloy, Brian Mockford, Seamus O’Hagan, David Beverland

**Affiliations:** 1 Primary Joint Unit, Musgrave Park Hospital, Belfast Health and Social Care Trust, Stockman’s Lane, Belfast, BT9 7JB UK; 2 Theatres, Musgrave Park Hospital, Belfast Health and Social Care Trust, Stockman’s Lane, Stockman’s Lane, Belfast, BT9 7JB UK; 3 Department of Anaesthesia, Ulster Hospital, Upper Newtownards Road, Dundonald, Belfast, BT16 1RH UK; 4 Department of Haematology, Tower block, Belfast City Hospital, Belfast Health and Social Care Trust, 51 Lisburn Road, Belfast, BT9 7AB UK; 5 Pharmacy Department, The Royal Hospitals, Belfast Health and Social Care Trust Grosvenor Road, Belfast, BT12 6BA UK; 6 Northern Ireland Clinical Trials Unit (NICTU), The Royal Hospitals, 1st Floor Elliott Dynes Building, Grosvenor Road, Belfast, BT12 6BA UK

**Keywords:** Tranexamic acid, Blood loss, Hip, Knee, Arthroplasty, Replacement, Post-operative, Oral

## Abstract

**Background:**

While it is has been proven that tranexamic acid (TXA) reduces blood loss in primary total hip and knee arthroplasty (THA and TKA), there is little published evidence on the use of TXA beyond 3 h post-operatively. Most blood loss occurs after wound closure and the primary aim of this study is to determine if the use of oral TXA post-operatively for up to 24 h will reduce calculated blood loss at 48 h beyond an intra-operative intravenous bolus alone following primary THA and TKA. To date, most TXA studies have excluded patients with a history of thromboembolic disease.

**Methods/design:**

This is a phase IV, single-centred, open-label, parallel-group, randomised controlled trial. Participants are randomised to one of three groups: group 1, an intravenous (IV) bolus of TXA peri-operatively plus oral TXA post-operatively for 24 h; group 2, an IV bolus of TXA peri-operatively or group 3, standard care (no TXA). Eligible participants, including those with a history of thromboembolic disease, are allocated to these groups with a 2:2:1 allocation ratio. The primary outcome is the indirectly calculated blood loss 48 h after surgery. Researchers and patients are not blinded to the treatment; however, staff processing blood samples are. Originally 1166 participants were required to complete this study, 583 THA and 583 TKA. However, following an interim analysis after 100 THA and 100 TKA participants had been recruited to the study, the data monitoring ethics committee recommended stopping group 3 (standard care).

**Discussion:**

TRAC-24 will help to determine whether an extended TXA dosing regimen can further reduce blood loss following primary THA and TKA. By including patients with a history of thromboembolic disease, this study will add to our understanding of the safety profile of TXA in this clinical situation.

**Trial registration:**

ISRCTN registry, ISRCTN58790500. Registered on 3 June 2016, EudraCT: 2015–002661-36.

**Electronic supplementary material:**

The online version of this article (10.1186/s13063-018-2784-3) contains supplementary material, which is available to authorized users.

## Background

Increased peri-operative and post-operative blood loss following total hip arthroplasty (THA) and total knee arthroplasty (TKA) can significantly influence the morbidity and mortality for both procedures [1]. Expected blood loss in these operations can range between 500 and 1600 mL [2, 3]. The resulting anaemia increases a patient’s risk of cardiovascular and respiratory complications, increases infection rates and impedes functional mobility.

Tranexamic acid (TXA) is well established in many areas of medicine for reducing blood loss [4] and is a mainstay in the management of traumatic haemorrhage in the emergency department [5, 6]. In lower limb arthroplasty, two meta-analyses of randomised controlled trials using TXA in THA and TKA concluded in favour of its use to limit blood loss [7, 8]. TXA in elective arthroplasty is not yet supported by the National Institute for Health and Care Excellence or British Orthopaedic Association guidelines. In part, this is because, despite a lack of published evidence, there are theoretical concerns about the potential increased risk of venous thromboembolism (VTE) with TXA [9]. Other countries, such as the USA, have embraced the use of TXA in arthroplasty [10–12], favouring the proven benefit of reduced blood loss in these typically elderly patients over the theoretical risk of VTE. To date, there have been no published safety concerns with this approach. A further issue with widespread TXA adoption is the absence of an established standard treatment regime in elective joint arthroplasty. As such, published studies describe a range of doses, routes of administration and durations of treatment [13–17].

Since the study protocol was first authorised for this trial in 2015, a wealth of studies has been published on TXA use in THA and TKA further advocating its use. However, determination of the most effective duration of TXA treatment, dosage and regimen for major joint arthroplasty has barely been investigated. Most studies focus on the intra-operative period [18, 19] with little published work into treatment regimes that extend beyond a dose 3 h post-operatively. However, we postulate that the period of greatest importance is the first 24 post-operative hours. This theory is based upon an internal audit carried out at Musgrave Park Hospital, Belfast, Northern Ireland, on 361 consecutive patients undergoing THA. We compared patients’ intra-operative blood loss to their total blood loss at 2 days post-operatively. Total blood loss was calculated indirectly using equations based on a change in haematocrit [20, 21]. The average directly measured intra-operative blood loss was 223 mL (range 50–1204 mL) and the average indirectly calculated total blood loss was 1377 mL (range 263–3864 mL). On average, the total loss was over six times the measured loss in theatre. Other relevant findings from this audit were that on average 90% of total loss occurs in the first 24 post-operative hours and that intra-operative blood loss grossly underestimates total blood loss, thus making it a poor predictor of total loss. Based on this audit and other literature demonstrating that blood loss continues in the first 24 h post-operatively [22–26], we hypothesise that using TXA up to 24 h post-operatively has the potential to reduce blood loss further following THA and TKA, compared to a single peri-operative dose of TXA. Since there is evidence that oral administration is equally efficacious as intravenous administration [27, 28], this study uses oral TXA post-operatively as a cheaper and less labour-intensive mode of delivery than either IV bolus or infusion regimes.

TRAC-24 will help to define the optimum treatment protocol for TXA use for peri-operative blood loss management in lower limb arthroplasty and will inform the standard of care both at the study site and elsewhere in relation to the use of peri-operative TXA. TRAC-24 will also contribute to the evidence base regarding TXA use and the risk of peri-operative venous and arterial thromboembolism. However, to determine definitively what impact TXA has on VTE in the peri-operative period, a larger-scale multi-centre study would be required.

### Objectives

The primary objective is to determine if the use of oral TXA post-operatively for up to 24 h will confer a reduction in calculated blood loss at 48 h beyond an intra-operative intravenous bolus alone for patients undergoing unilateral primary THA or TKA.

The secondary objective is to determine if the addition of oral TXA post-operatively to an intra-operative intravenous bolus of TXA produces any change in other measurable parameters compared to those observed for either an intra-operative intravenous bolus alone or no TXA for patients undergoing unilateral primary THA or TKA.

## Methods/design

### Study design

**Fig. 1:**
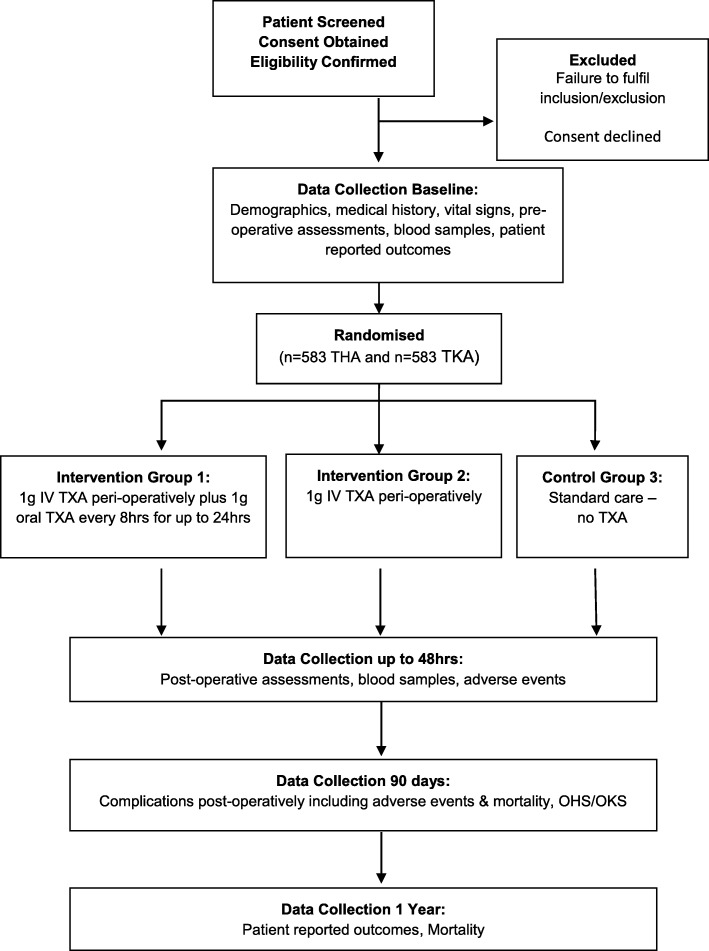
Flowchart for study participants. IV intravenous, OHS Oxford hip score, OKS Oxford knee score, Op operative, TXA tranexamic acid

**Fig. 2:**
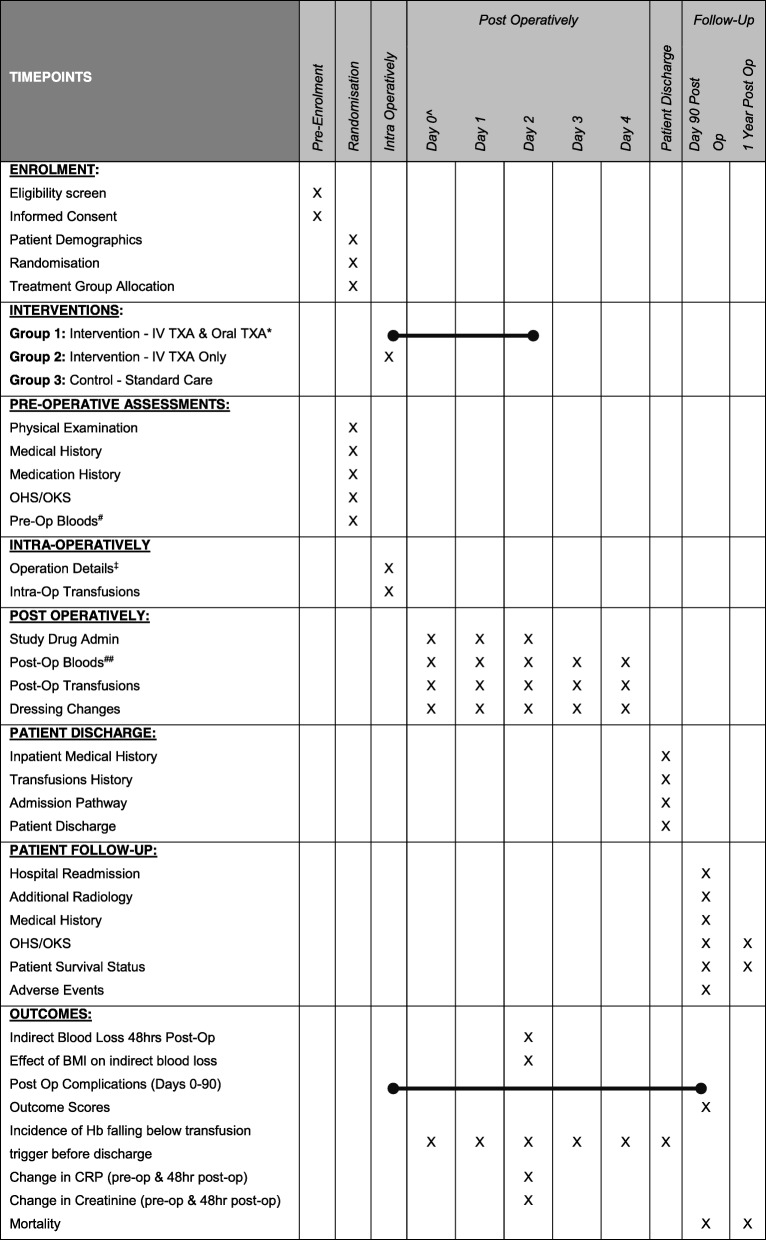
Schedule of enrolment, intervention and assessments. CRP C-reactive protein, Hb haemoglobin, Hct haematocrit, IV intravenous, INR International normalised ratio, KTS knife to skin, OHS Oxford hip score, OKS Oxford knee score, TXA tranexamic acid

This is a phase IV, single-centred, open-label, parallel-group, randomised controlled trial assessing superiority. Elective primary THA and TKA patients are allocated to either one of two TXA intervention groups or one non-treatment comparator group. A study flowchart is shown in Fig. 1. The protocol was written in accordance with the Standard Protocol Items: Recommendations for Interventional Trials (SPIRIT). A SPIRIT figure can be seen in Fig. 2. The SPIRIT checklist is given in Additional file 1.

When the study was initially planned, the peri-operative use of TXA was not a standard of care in primary THA and TKA in the UK and local site policy reserved its use for patients considered at high bleeding risk. Therefore, the no-intervention control group was deemed necessary to reflect current practice. However, following an interim analysis and review of safety data, which was performed as part of a scheduled data monitoring and ethics committee (DMEC) meeting, it was recommended that randomisation to control group 3 be discontinued. This DMEC meeting took place after 200 participants (40 of whom were in control group 3) had been recruited. By the time approval was gained for this protocol change, 134 participants had been recruited to the control group.

### Primary outcome measure

The primary outcome measure is the total calculated volume of blood loss, in millilitres, at 48 h. This is calculated indirectly, from a series of equations [20, 21, 29].

The first equation determines the patient’s blood volume in litres:

$$ \mathrm{PBV}=\left({k}_1\times {h}^3\right)+\left({k}_2\times w\right)+{k}_3, $$

where

PBV = patient’s blood volume (L),

*h* = height (m),

*w* = weight (kg),

*k*_1_ = 0.3669 for men and 0.3561 for women,

*k*_2_ = 0.03219 for men and 0.03308 for women

and

*k*_3_ = 0.6041 for men and 0.1833 for women.

The second equation determines the volume of red blood cells lost in the period from immediately before the operation to 48-h post operatively. Note that PBV should be converted to millilitres prior to calculation of RBC.

$$ \mathrm{RBC}=\mathrm{PBV}\times \left({\mathrm{Hct}}_{\mathrm{pre}\hbox{-} \mathrm{op}}-{\mathrm{Hct}}_{\mathrm{post}\hbox{-} \mathrm{op}48\mathrm{h}}\right), $$

where

RBC = red blood cell volume loss (mL),

Hct_pre-op_ = pre-operative haematocrit

and

Hct_post-op 48h_ = post-operative haematocrit.

The third and final equation converts red cell volume loss into whole blood volume loss. We selected the Gross formula for this [29] and adapted it to incorporate transfusions as per the Mercuriali formula [21].

To convert red blood cell volume loss into total blood volume loss, the RBC must be divided by the average Hct. In addition, volume of transfused blood must be taken into account. An average unit of blood has a volume of 280ml with an Hct of 0.6, which is the red cell equivalent of 168mls with an Hct of 1.0. As this is diluted by patient blood volume, the value must again be divided by average Hct.

The Gross formula (adapted assuming an average Packed Red Cells (PRC) unit is 280 mL with a Hct of 0.6) is

$$ \mathrm{Total}\kern0.5em \mathrm{volume}\kern0.5em \mathrm{loss}=\frac{\mathrm{RBC}}{{\mathrm{Hct}}_{\mathrm{avg}}}+\left(\left(\mathrm{No}\kern0.5em \mathrm{units}\kern0.5em \mathrm{PRCs}\times 168\right)/{\mathrm{Hct}}_{\mathrm{avg}}\right), $$

where

$$ {\mathrm{Hct}}_{\mathrm{avg}}=\frac{\left({\mathrm{Hct}}_{\mathrm{pre}\hbox{-} \mathrm{op}}+{\mathrm{Hct}}_{\mathrm{post}\hbox{-} \mathrm{op}48\mathrm{h}}\right)}{2}. $$

The resultant total volume loss is the primary outcome measure. Traditionally, the primary outcome measure in TXA studies has been transfusion rate. The incidence of transfusion at the study site for TKA patients reduced from 76.0% in 2001 to 5.8% in 2013. This fall preceded TXA use and was a consequence of a more restrictive transfusion policy. Consequently, because transfusion rates are now relatively low, transfusion was not considered to be the most relevant primary outcome measure. As the primary outcome measure, total volume loss is independent of clinician or investigator bias, effectively becoming a blinded outcome measure. Transfusion is more vulnerable to bias because although a transfusion trigger is set prior to surgery, it is simply a prompt for clinicians to consider transfusion and not necessarily to transfuse. Essentially fit patients <65 years have a trigger of <70 g/L and those >65 a trigger of <80 g/L. All those with potential risk factors have a trigger of <90 g/L and symptomatic patients a trigger of <10 g/L.

The study also has a number of secondary and exploratory outcomes. The secondary outcomes characterise other benefits of the interventions for patients undergoing primary THA or TKA. Exploratory outcome measures will not be evaluated to the same extent as the primary and secondary outcomes but have the potential to generate future studies.

### Secondary outcome measures

The secondary outcomes are:

- Incidence of post-operative haemoglobin (Hb) falling below the transfusion trigger (irrespective of transfusion) prior to discharge
- Effect of body mass index on the volume of indirect blood loss at 48 h post-surgery
- Change in C-reactive protein (CRP) level from pre-operative to 48 h post-operative
- Change in creatinine level from pre-operative to 48 h post-operative
- 90-day mortality
- year mortality

### Exploratory outcome measures

Exploratory outcomes include:

- Comparison of intra-operative blood loss between the groups
- Number of wound dressing changes in the first 48 h post-operatively
- Requests (and results) for post-operative troponin levels prior to discharge
- Post-operative length of hospital stay
- Incidence of allogenic blood transfusion prior to discharge
- Incidence of allogenic blood transfusion within 90 days of surgery
- Incidence of post-operative Hb falling below the transfusion trigger without the patient being transfused to time of discharge
- Incidence of post-operative arrhythmia within 90 days of surgery
- Incidence of s within 90 days of surgery
- Incidence of proximal deep vein thrombosis within 90 days of surgery
- Incidence of myocardial infarction within 90 days of surgery
- Incidence of cerebrovascular accidents within 90 days of surgery
- Emergency hospital admission or unplanned critical care admission within 90 days of surgery
- Number of returns to theatre for wound problems within 90 days of surgery
- Difference in indirect blood loss at 48 h between patients on aspirin as a VTE prophylaxis compared with those on enoxaparin
- Difference in indirect blood loss at 48 h between patients placed in flexion in a knee jig for 6 h post-operatively as opposed to those placed in flexion on a pillow
- Difference in indirect blood loss at 48 h between patients for whom a tourniquet was used compared to those for whom a tourniquet was not used during TKA
- Change in Oxford hip score (OHS) or Oxford knee score (OKS) from pre-operatively to 90 days post-operatively
- Change in OHS or OKS from pre-surgery to 1-year post-surgery
- Differences in hospital costs associated with each treatment group

The OHS and OKS are outcome measures [30] widely used pre- and post-operatively for all patients at the study site to measure the success of an operation. It will be important to ensure that there are no differences in OHS/OKS at 1 year between each of the groups. The OKS/OHS is assessed for patients by hospital staff at their pre-operative assessment clinic, at the 90 day follow-up review clinic (for TKA patients only) and at the patient’s 1-year clinic as part of standard care. Patients must attend their pre-operative assessment clinic, but should any patient be unable to attend a post-operative clinic, the OHS/OKS will be completed via telephone. THA patients have an early post-operative clinic at 6 weeks routinely, so for this study the 90-day OHS is assessed over the telephone by research nurses. Because the participants are not blinded to their intervention, there is the potential for bias when completing the Oxford scores at 90 days and 1 year following surgery. Practically, we feel any such effect would be negligible.

The incidence of death and complications at 90-days post-operatively are clinically important outcomes for this study. While this study is not powered to answer whether the use of TXA can lower complications, it is important that complications are reported.

### Study setting

This is a single-centre study taking place in a Primary Joint Unit in a regional orthopaedic centre in the UK with patients under the care of one of four consultant orthopaedic surgeons being recruited to the study.

### Study participants

Participants who are on the waiting list for a primary THA or TKA within the Primary Joint Unit are screened for study inclusion.

### Inclusion criteria

The following inclusion applies:

- Awaiting primary elective THA or TKA
- ≥18 years and ≤100 years of age

### Exclusion criteria

The exclusion criteria for this trial are:

- Do not pass a pre-operative assessment for elective THA or TKA
- Fractured neck of femur
- Haemophiliac or coagulation disorders that require TXA
- Allergy to TXA or any of its excipients
- Platelets less than 75,000/mm^3^ at pre-operative assessment*
- Active treatment for deep VTE or pulmonary embolism within 6 months prior to surgery*
- History of VTE within 6 months prior to surgery*
- Myocardial infarction within 12 months prior to surgery*
- Cardiac stent within 12 months prior to surgery*
- Cerebrovascular accident or transient ischemic attack within 9 months prior to surgery*
- Use of antiplatelet medication within 7 days prior to surgery* (does not include aspirin if dose <300 mg).
- Direct thrombin inhibitors within 2 days prior to surgery*
- Factor Xa inhibitors within 2 days prior to surgery*
- The international normalised ratio is greater than or equal to 1.5 in a patient who has stopped warfarin in preparation for surgery
- Hepatic failure*
- History of epilepsy
- Requiring therapeutic anticoagulation post-operatively, e.g. due to a metallic heart valve
- Pregnant women, women who have not yet reached the menopause (no menses for ≥12 months without an alternative medical cause) who test positive for pregnancy or are unwilling to take a pregnancy test prior to trial entry
- Have been using combined hormonal contraception (which includes combined oral contraception, a combined contraceptive transdermal patch and a vaginal ring) within 4 weeks prior to surgery*
- Female patients who are breastfeeding
- Treated with any other investigational medication or device within 60 days prior to surgery
- Unable to provide informed consent
- Unable or unwilling to commit to the study schedule of events
- Unwilling to provide informed consent
- Present for simultaneous bilateral THA or TKA
- On renal dialysis and have an arteriovenous fistula
- Have previously been enrolled in this study

*These patients at the study site have contra-indications to primary THA/TKA.

### Recruitment and consent

TRAC-24 originally required a minimum of 1166 patients to be recruited. Recruitment would stop when 583 participants were recruited for both TKA and THA. However, following a DMEC recommendation, recruitment to control group 3 was stopped, after approval from the sponsor, the regional ethics committee and the Medicines and Healthcare Products Regulatory Agency (MHRA) was acquired. At this point, 672 participants had been recruited to the study. This meant that a minimum of 466 THA and 466 TKA were now required to complete the study with a minimum overall recruitment of 1066, recruiting only to groups 1 and 2. Potentially eligible patients are identified and screened by members of the clinical study team based on the inclusion and exclusion criteria outlined above.

A cover letter and a patient information sheet approved by the Research Ethics Committee is sent to potential patients informing them about the study. The latter contains a contact number should they have any questions or wish to discuss the study further. The study is then discussed directly with the patient at a pre-operative clinic (approximately 7 to 10 days prior to operation) or following admission for surgery. Informed consent is taken by a member of the research team after the participant has been given the opportunity to ask further questions and agrees to participate.

### Study assessments

All patients are evaluated during the study according to the schedule of enrolment, interventions and assessments as outlined in Fig. 2. Data are collected at each of the following time points: pre-operatively, intra-operatively, post-operatively until discharge and then at 90 days and 1 year post-operatively.

### Randomisation and blinding procedures

**Fig. 3:**
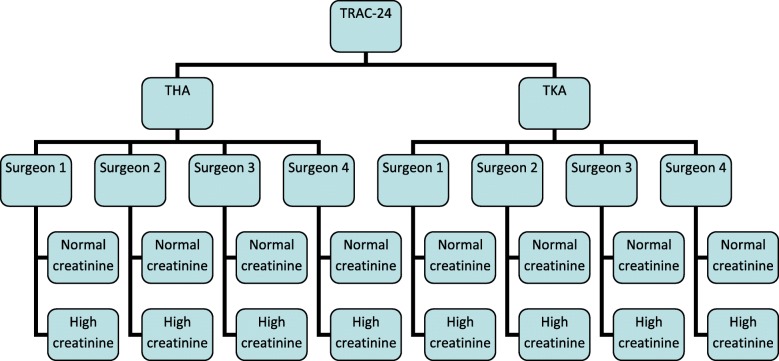
Stratification factors. THA total hip arthroplasty, TKA total knee arthroplasty

At the outset of the trial, eligible participants were allocated to intervention group 1, intervention group 2 or control group 3 (standard care) with an allocation ratio of 2:2:1. After the DMEC recommended stopping randomisation to control group 3, eligible participants were then allocated to intervention group 1 or intervention group 2 with an allocation ratio of 1:1. Blocked randomisation with randomly permuted block sizes is used. Participants are stratified according to operation type, surgeon and creatinine level, resulting in 16 possible stratifications (Fig. 3):

The randomisation sequence is concealed using sealed envelopes with tamper-proof labels. The trial statistician generated the randomisation sequence. When a participant has been recruited, consented and admitted to the ward pre-operatively, a sealed envelope is opened in sequence according to the stratification. Two members of the research team must be present for this. The envelope is not opened until the post-operative transfusion trigger has been recorded, which is set after consent and prior to randomisation. Anaesthetists, surgeons, and other theatre, recovery and ward staff will not be blinded to the treatment, nor will the study investigators or the patient themselves. The decision to transfuse is not blinded but the transfusion trigger is blinded. This means that the number of patients falling below that transfusion trigger is blinded. Laboratory staff who process the blood samples are blinded to the treatment allocation.

### Interventions

For intervention group 1, intra-operative intravenous TXA is administered within 30 min before knife to skin (KTS) or application of a tourniquet plus oral TXA at 2 h, 10 h, 18 h and 26 h post KTS. Each oral dose should be given within a 2 h window but not earlier than the planned time point for the first dose. Patients with renal impairment will receive a reduced dose dependent on their pre-operative serum creatinine as per Table 1.

**Table 1:** Study drug administration

	Up to 30 mins pre KTS	+ 2 h post KTS	+ 10 h post KTS	+ 18 h post KTS	+ 26 h post KTS
Intervention group 1 Normal creatinine <120 μmol/L	1 g TXA IV	1 g TXA oral	1 g TXA oral	1 g TXA oral	1 g TXA oral
Intervention group 1 High (A) creatinine level 120–249 μmol/L	0.5 g TXA IV	0.5 g TXA oral	0.5 g TXA oral	0.5 g TXA oral	0.5 g TXA oral
Intervention group 1 High (B) creatinine level 250–499 μmol/L	0.5 g TXA IV	–	0.5 g TXA oral	–	0.5 g TXA oral
Intervention group 1 High (C) creatinine level ≥ 500 μmol/L	0.5 g TXA IV	–	–	–	0.5 g TXA oral
Intervention group 2 Normal creatinine level < 120 μmol/L	1 g TXA IV	–	–	–	–
Intervention group 2 High (A) creatinine level 120–249 μmol/L	0.5 g TXA IV	–	–	–	–
Intervention group 2 High (B) creatinine level 250–499 μmol/L	0.5 g TXA IV	–	–	–	–
Intervention group 2 High (C) creatinine level ≥ 500 μmol/L	0.5 g TXA IV	–	–	–	–
Control group 3 – no TXA All creatinine levels	–	–	–	–	–

*IV* intravenous, *KTS* knife to skin, *TXA* tranexamic acid

For intervention group 2, intra-operative intravenous TXA is administered within 30 min before KTS or application of a tourniquet. Patients with renal impairment will receive a reduced dose dependent on their pre-operative serum creatinine.

### Study drug

TXA 100 mg/mL solution for injection and TXA 500 mg tablets are regarded as investigational medicinal products for this study.

The study medication is provided as:

- Packs containing two 5-mL ampoules of TXA 100 mg/mL solution for injection
- Packs containing one 5-mL ampoule of TXA 100 mg/mL solution for injection (for patients with renal impairment)
- Packs containing eight TXA 500 mg tablets
- Packs containing four tablets, two tablets or one tablet TXA 500 mg (for patients with renal impairment)

Packs are labelled with a unique medication pack identification number in accordance with applicable regulatory requirements. On receipt of a study medication request form, signed by an authorised member of the research team, the pharmacy department supplies a number of medication packs to the operating theatre. These are then available for administration to patients according to the treatment allocation specified in the assigned sealed envelope. The medication pack goes with the patient from theatre to ward depending on randomisation.

Drug administration is recorded on the patient’s hospital prescription chart and on their case report form (CRF).

Used and unused medication packs are returned to the site pharmacy department. The site pharmacist is responsible for maintaining complete records of the disposition of all study drugs that are received, dispensed, unused, expired, returned to pharmacy or destroyed. The Northern Ireland Clinical Trials Unit (NICTU) provides a drug accountability record form for this purpose. All investigational medicinal products for this study are sourced, packed and labelled for the trial by Victoria Pharmaceuticals, Belfast Health and Social Care Trust.

TXA is administered by slow intravenous injection or orally at the time points indicated in Table 1 in accordance with the treatment allocation specified in the assigned sealed envelope. There are no relevant concomitant care and interventions that are permitted or prohibited during the trial.

### Safety

The conduct of the trial is overseen by a trial steering committee (TSC). Throughout the trial, the TSC takes responsibility for monitoring and guiding overall progress, scientific standards, operational delivery and protecting the rights and safety of trial patients. Monthly trial management group meetings are held involving the NICTU and members of the research team. An independent DMEC was appointed with responsibility for safeguarding the interests of trial patients. This committee monitors the main outcome measures including safety and efficacy. It also assists and advises the TSC to protect the validity and credibility of the trial. Charters for the TSC, DMEC and the trial management group are held by the NICTU.

The principal investigator or designee records all directly observed adverse events (AEs) and all AEs spontaneously reported by the patient. The principal investigator or designee assesses all AEs for seriousness, causality and severity, and if the AE is related to the study drug, for expectedness.

The AE reporting period for the participant begins upon enrolment into the trial and ends 30 days following the last administration of the study drug. All AEs are assessed by the principal investigator as possibly, probably or definitely related to the study drug and all serious adverse events (SAEs) that occur during this time will be followed until they are resolved or are clearly determined to be due to a patient’s stable or chronic condition or intercurrent illness(es). Reporting follows the regulatory requirements and NICTU standard operating procedures.

For TRAC-24, a SAE is an AE that:

- results in death
- is life-threatening
- requires hospitalisation or prolongation of existing hospitalisation (even if only for 1 day)*
- results in persistent or significant disability or incapacity
- involves a congenital anomaly or birth defect
- is any other important medical events that carries a real, not hypothetical, risk of one of the outcomes above.

*Hospitalisation is defined as an inpatient admission regardless of length of stay, even if the hospitalisation is a precautionary measure for continued observation. Hospitalisations for a pre-existing condition, including elective procedures that have not worsened, do not constitute an SAE.

### Data collection and management

An electronic CRF is used to collect the data for each study subject. Patient identification on the CRF is through their unique trial identifier, allocated at the time of recruitment. Data coding is carried out at various stages during the study. The electronic CRF is subject to audit by the sponsor. Data integrity and study credibility depend on factors such as ensuring adherence to the protocol and using quality control measures to establish and maintain high standards for data quality. To ensure accurate, complete and reliable data are collected, the NICTU provide training to site staff in the format of investigator meetings and/or site initiation visits. The data are validated and discrepancy reports are generated following data entry to identify discrepancies, such as out of range values, inconsistencies or protocol deviations based on data validation checks programmed into the clinical trial database. Data security and storage follows the data management plan specific to this study, NICTU standard operating procedures and the sponsor’s requirements. The DMEC is convened to carry out reviews of the data at staged intervals during the study.

The dropout rate is expected to be negligible with little or no missing data for the primary outcome measure. Standard approaches will be used to detect patterns in missing data for the other outcomes.

### Statistical and interim analysis

Based on a mean (standard deviation) of 1225 (499) mL of blood loss and a clinically significant difference of 150 mL (12%) between the two TXA groups, originally the trial required 233 participants per group at 90% power and 0.05 level of significance. Due to the proven greater blood loss in control group 3, there were only half this number of participants in this group (*n* = 117). This would result in a total of 1166 participants (583 TKA and 583 THA), as a negligible dropout rate was expected. The clinically significant difference of 150 mL was based on an internal audit of blood loss in primary THA and TKA patients. The audit found that the average calculated blood loss was almost 1500 mL. Based on this and the clinical opinion of the anaesthetists, surgeons, and haematologist involved in the study, a value of 150 mL (10% of the average blood loss) was considered to be clinically significant.

Based on the strong rationale that TXA is effective at reducing blood loss for THA and TKA, a planned interim analysis was performed to compare the control and combined intervention groups to determine whether recruitment to control group 3 should continue. A power calculation determined that a sample size of 98 patients would be required to detect a clinically relevant difference of 450 mL of blood loss between the control and combined intervention groups at the significance level of *p* = 0.01 and 90% power. The analysis was performed separately for THA and TKA participants and also for the combined participants.

Following this interim analysis, the DMEC recommended to the TSC that randomisation to control group 3 of patients recruited should be stopped. A minimum of 932 patients is required with an allocation of 466 (233 TKA and 233 THA) to intervention group 1 and 466 (233 TKA and 233 THA) to intervention group 2. Atogether, 134 patients were randomised to control group 3. Eligible participants are now allocated to intervention group 1 or intervention group 2 with an allocation ratio of 1:1.

Analyses will be on an intention-to-treat basis and at an a priori significance level of *p* = 0.05. Baseline characteristics and outcome measures will be summarised as mean and standard deviation, median and inter-quartile range or numbers and proportions (%) depending on the scale of measurement. For the primary outcome and other continuous outcomes, analysis of variance with contrasts will be used to compare both intervention groups and also the intervention groups combined versus the control group. Analysis of covariance will be used to adjust for baseline and other covariates where appropriate. The chi-squared test will be used to test the difference in the proportions between the groups for the categorical variables. All analyses will be performed for THA and TKA separately and also combined. Subgroup analyses will be performed on the primary outcome measure for THA/TKA, creatinine, age, gender and body mass index. A statistical analysis plan will be written and approved prior to commencement of any analyses.

### Health economic analysis

A cost analysis will be performed from a hospital perspective to compare the cost per patient in each treatment group. Data relating to patients’ primary hospital admission is collected prospectively until primary hospital discharge (including but not limited to their length of stay on a ward and in the intensive care unit). Data relating to readmissions, accident and emergency attendances and outpatient attendances is collected at 90 days. Unit costs will be obtained from public sources, e.g. Department of Health reference costs. Differences in costs between groups will be estimated using regression methods adjusted for baseline and other covariates where appropriate.

## Discussion

TXA is effective at reducing blood loss in elective joint arthroplasty. TRAC-24 extends administration of TXA to primary THA and TKA patients up to 24 h post-operatively with the aim of further reducing blood loss. The coagulation pathway for TXA is a system with a tightly controlled balance and theoretically TXA could act as a pro-coagulant and produce VTE. To date, there is no evidence to suggest it does. A Cochrane review in 2011 concluded that TXA did not increase the risk of death, myocardial infarction, cerebrovascular accident, deep vein thrombosis, pulmonary embolism or renal impairment [31]. In 2014, Poeran retrospectively analysed outcome data on 872,416 TKA or THA patients and found no increase in thromboembolic events nor renal impairment with TXA use [32]. However, all published randomised controlled trials to date have excluded patients with previous thromboembolic disease. The data are, thus, criticised for not reflecting a true safety profile. TRAC-24 is novel in that such patients are not excluded and thus, it is intended that this study will assist in defining the safety profile of TXA in primary joint arthroplasty for patients with a history of thromboembolic disease.

Since TRAC-24 commenced, there have been several challenges to overcome. Firstly, recruitment has taken longer than expected. This is primarily because of a reduction in the number of primary THA and TKA operations performed at the study site since the study was planned. In addition, there was a delay in starting the study and we have also found seasonal changes in recruitment due to, for example, a winter bed crisis that reduced the number of elective procedures performed. Additional funding was secured to enable the study to continue. In addition, there was difficulty in collecting some of the data for the exploratory outcomes at the 90-day time point. The protocol stated that participants would be contacted via telephone for this information; however, the information they provided was not always complete and participants did not always answer their telephone. To avoid important safety data being missing for the study, an amendment was approved allowing us to use the Northern Ireland Electronic Care Record to collect this data for all prospective participants and for all participants who had already been recruited, if their data could not be obtained using the original protocol.

Originally, this study had been designed with three arms to include both interventions and also a control arm, which was standard care without TXA. The numbers allocated to each arm were in the ratio 2:2:1. It was felt necessary to include the no TXA arm, control group 3, because although TXA is known to reduce blood loss, it was not the standard of care in our unit. However, it was stipulated in the protocol that there must be an interim analysis once 100 THA and 100 TKA patients had been recruited to decide on the continuation of the control group 3. At that time, 40 patients had been recruited into this group. Following this analysis, the DMEC recommended dropping control group 3, which was ratified by the TSC. This change was implemented following approvals from the sponsor, the research ethics committee and MHRA. This also resulted in a change in practice in our unit, with TXA being considered for all primary joint patients at the discretion of the anaesthetist.

The authors are aware of some limitations to this study. Firstly, it is a single-centre study; therefore, it could be argued that the results are not transferable to other centres due to different clinical practices. Secondly, randomisation is performed using sealed envelopes with tamper-proof labels instead of an automated system. While both systems were considered during the study design, it was decided that as it was a single-centre study with robust checks by the study site and by the NICTU, sealed envelopes were sufficient.

With TRAC-24, we intend to determine if the effect of TXA can be potentiated by continuing its administration post-operatively, at the time of greatest loss.

### Trial status

Recruitment started in July 2016 and is ongoing at the time of manuscript submission. At the end of February 2018, 879 participants have been recruited to the study and protocol version 5 (8 November 2017) is currently in use. As with previous amendments, changes to the protocol and any related documents (e.g. the patient information sheet) require regulatory authority or ethics committee approval prior to implementation, except when modification is needed to eliminate an immediate hazard to patients. It is expected that the study will be fully recruited by July 2018.

## Additional file

Additional file 1: SPIRIT 2013 Checklist: Recommended items to address in a clinical trial protocol and related documents. (DOC 137 kb)

## Abbreviations

AE: Adverse event
AR: Adverse reaction
BOIS: Belfast Orthopaedic Information System
CI: Chief investigator
CREST: Clinical Resource Efficiency Support Team
CRF: Case report form
CRP: C-reactive protein
DMEC: Data monitoring and ethics committee
DOS: Day of surgery
Hb: Haemoglobin
Hct: Haematocrit
INR: International normalised ratio
IV: Intravenous
KTS: Knife to skin
MHRA: Medicine and Healthcare Products Regulatory Agency
NICTU: Northern Ireland Clinical Trials Unit
OHS: Oxford hip score
OKS: Oxford knee score
PBV: Patient’s blood volume
RBC: Red blood cell volume loss
SAE: Serious adverse event
THA: Total hip arthroplasty
TKA: Total knee arthroplasty
TSC: Trial steering committee
TXA: Tranexamic acid
VTE: Venous thromboembolism
